# Comparing adverse maternal and perinatal outcomes in primary caesarean delivery during first versus second-stage of labour in Kenya: An institution-based cohort study

**DOI:** 10.1371/journal.pone.0294266

**Published:** 2023-11-27

**Authors:** Kimbley Asaso Omwodo, Edwin Were

**Affiliations:** Department of Reproductive Health, Moi University, School of Medicine, Eldoret, Kenya; Kasr Alainy Medical School, Cairo University, EGYPT

## Abstract

**Background:**

As caesarean delivery rates continue to increase globally, so are the number of second-stage caesarean deliveries. Second-stage caesareans may carry additional risk of complications for both the mother and fetus owing to fetal head impaction into the maternal pelvis and manipulations required for delivery. So far, data on this procedure’s outcomes from low resource countries are limited.

**Objectives:**

To compare adverse maternal and perinatal outcomes between second-stage and first-stage of labour intrapartum primary caesarean deliveries over 12 months at a tertiary referral obstetric hospital in Kenya.

**Methods:**

In a hospital-based cohort study, 222 women with singleton, cephalic presenting fetuses at term gestation who had intrapartum primary caesarean delivery during active labour were recruited post-partum. Second-stage caesarean deliveries (73) were compared to 149 first-stage caesarean deliveries. The proportion of caesarean deliveries in the second-stage of labour was estimated and the adverse maternal and perinatal outcomes were compared. The study was conducted from August 2021 to July 2022 at the Moi Teaching and Referral Hospital, Eldoret.

**Results:**

The proportion of second-stage caesarean deliveries among intrapartum primary caesarean deliveries was 4.3% [95% CI: 2.9% - 4.7%]. Compared to first-stage caesarean deliveries, second-stage caesarean deliveries had a significantly higher risk of adverse maternal outcomes (RR 3.272, 95% CI 2.28–4.71, P < 0.001), including intraoperative trauma, atony, blood transfusion, and a postoperative hospital stay of more than three days. Additionally, there was a higher risk of adverse perinatal outcomes (RR 2.748, 95% CI 2.45–4.50, P < 0.001), including increased risk of a 5-min APGAR ≤3, admission to NBU, and neonatal death.

**Conclusions:**

An increased risk of adverse maternal and perinatal outcomes is associated with primary second-stage caesarean deliveries compared to primary first-stage caesarean deliveries.

## Background

Caesarean delivery is a vital intervention for women when complications occur during pregnancy and labour. Pregnancy-related morbidity and mortality in these deliveries are higher (35.9 deaths per 100,000 live deliveries) compared to vaginal delivery (9.2 deaths per 100,000 live births), as well as potential risks in future pregnancies and long-term health [1,2].

The rising caesarean delivery rates are a global concern; in most countries, the incidence is well above what is expected based on obstetric indications. In Kenya, caesarean delivery rates have varied widely over the last decade, with an average rate of 11.6% in public healthcare settings and 38.1% in private facilities [3,4].

As caesarean delivery rates increase, so does the rate of second-stage caesarean deliveries, which may carry additional risk due to fetal head impaction and manipulations required to deliver the baby [5]. In lower and middle income countries, the practice of operative vaginal delivery is declining in usage, while second-stage caesarean delivery is progressively becoming a more prevalent alternative [6]. Lack of training and supervision for junior staff, loss of technique associated with difficult-assisted delivery, and concerns about maternal and neonatal morbidity and litigation all contribute to this trend [7].

Most studies on second-stage caesarean delivery are from high resource countries. The safety, outcomes, and complications of second-stage caesarean delivery are not well understood in low and middle-income (LMIC) economies. There is an absence of similar research in obstetric units in Kenya, resulting in a knowledge gap and challenges in assessing trends and comparing evidence.

This study serves as a baseline for potential interventions in second-stage delivery in LMIC. Understanding risks can inform clinicians’ decisions on pregnancy and delivery care at the local level and contribute to quality evidence on this emerging topic.

This study aimed to evaluate adverse maternal and perinatal outcomes in the second-stage of labour compared to the first-stage labour intrapartum primary caesarean deliveries at Moi Teaching and Referral Hospital (MTRH), Eldoret.

## Methods

This ambi-directional cohort study was conducted at the Riley Mother and Baby Hospital (RMBH) unit, part of the Moi Teaching and Referral Hospital in Eldoret, Kenya. As Kenya’s second largest public teaching and referral hospital, RMBH is a major tertiary health care centre. The hospital provides obstetric services, with an average of 12,000 deliveries per year and serves a population of approximately 24 million people from Western Kenya, parts of Eastern Uganda, and Southern Sudan.

The target population consisted of women who had undergone intrapartum primary caesarean delivery at the hospital between August 1, 2021, and July 31, 2022. The study’s inclusion criteria were singleton pregnancy, cephalic presentation, ≥37+0 gestation (Robson group 1,2a,3,4a) [8], and with intrapartum primary caesarean delivery performed during active phase of labour. The study’s exclusion criteria were determined based on potential confounders, including known major fetal abnormalities, and high-risk pregnancies (i.e., significant maternal disease or pregnancy complications such as hypertension, diabetes, or intrauterine growth restriction).

To better determine the average values of outcome data and minimise errors from testing a small number of possibly atypical samples, the study evaluated all eligible second-stage caesarean deliveries (exposed cases) carried out at the study site for 12 months.

All appropriate caesarean deliveries was allocated to one of three categories:

Group 1: Poor-progress labour/cephalopelvic disproportion/prolonged or obstructed labour/dystocia.

Group 2: Fetal distress/Non-reassuring fetal heart rate pattern/cord prolapse/ failed vacuum delivery.

Group 3: Other than groups 1 & 2.

Similar to Lurie et al., classification was done in order to retain enough statistical power to demonstrate clinically significant differences [9].

### Recruitment technique

Recruitment took place in the post-natal ward. The hospital obstetric procedure register was examined daily for caesarean deliveries performed in the preceding 24 hours. The obstetric procedure register contains information pertaining to patient name, in-patient number, previous ward of admission, indication for caesarean delivery and gestation at the time of caesarean delivery. The inpatient numbers of eligible women were traced to the post-natal ward. Both ’exposed’ and ’non-exposed’ women were recruited within 24hrs post caesarean procedure. Two ’non-exposed women’ were selected immediately after the ’exposure’ was identified in the same category of caesarean delivery indication (i.e. group 1, 2 or 3). "Exposed" cases were compared with two ’non-exposed’ participants per case considered representative of the cesarean delivery indication category.

A data abstraction form was used to collect the data. Participants’ hospital medical files were reviewed for retrospective data on intra-operative details, and immediate postoperative neonatal and maternal outcomes. Follow-up was done till hospital discharge for prospective data on maternal postoperative length of hospital stay outcome and 24-hour neonatal mortality.

The study variables were collected and measured, including independent variables such as maternal age, parity, gestational age, caesarean delivery indication, and the number of years of obstetric practice of the surgeons. The primary outcome was the composite of adverse maternal outcomes, while the secondary outcome was the composite of adverse perinatal outcomes. A composite measure was adopted to avoid an arbitrary choice between several important outcomes. This was particularly preferred for the rare outcome of mortality and the short study duration, as it increases statistical efficiency and reduces costs.

The adverse maternal outcome composite was defined as a woman experiencing any one or more of the following: intraoperative complications, primary post-partum haemorrhage, blood transfusion, Intensive care unit (ICU) admission or length of postoperative hospital stay >3 days. The adverse perinatal composite outcome was defined as a neonate experiencing any one or more of the following: neonatal trauma, New Born Unit (NBU) admission, Apgar score ≤7 at 5 min, or death within 24 hours of caesarean delivery.

The covariates in the study were demographic and obstetric characteristics, which included maternal age, parity, gestational age, fetal weight, caesarean delivery indication, and the number of years of obstetric practice of the surgeon.

The statistical analysis was performed using STATA version 15 SE. Differences in sociodemographic characteristics were assessed using chi-square tests for categorical variables and T-tests for continuous variables. Frequencies and relative risks (RR) were calculated for each component of the composite adverse maternal and perinatal outcomes. Regression analysis was planned for covariates with a p-value ≤ 0.05 and those of clinical importance. A significance level of P < 0.05 was considered statistically significant.

Statistical method for handling missing data: The mean value substitution method was used, replacing the missing value with the average value calculated over all the values available from the other waves of data collection.

No loss to follow-up occurred in this study, likely due to the short follow-up period limited to only the hospital stay.

Ethics approval and consent to participate Ethical clearance was obtained from the Moi University Institutional Research Ethics Committee (IREC)–Reference: IREC/2021/88—approval Number: 0003919. Permission from MRTH administration was also obtained. Written informed consent was taken from each subject before enrolment in the study following Good Clinical Practice (GCP) principles, and all methods were carried out in accordance with the Declaration of Helsinki. All data were maintained as confidential, and no individual was identified in the dissemination of findings.

## Results

**Fig 1:**
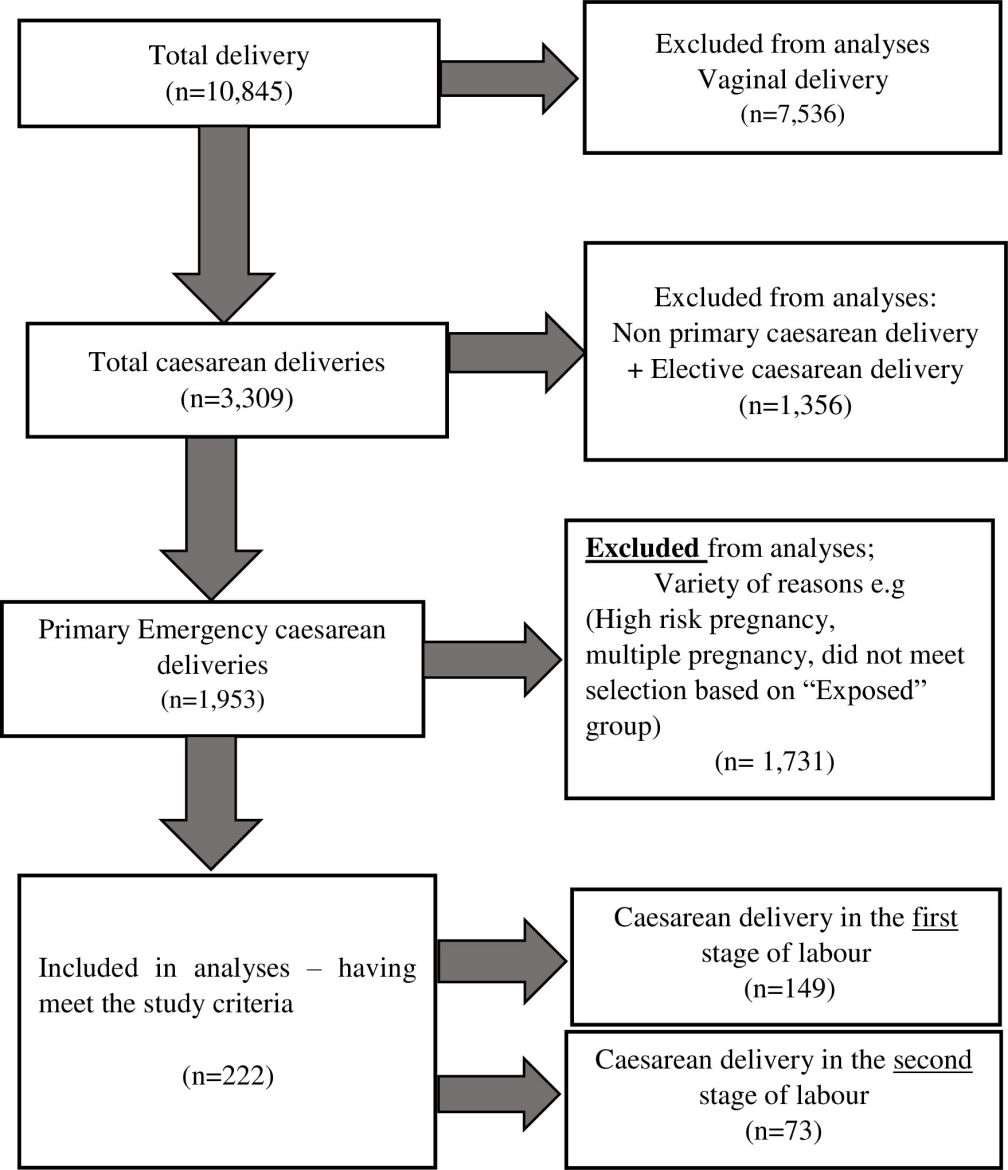
Modes of delivery during study period with case selection.

Over one year, the hospital recorded 10,857 deliveries, 30.5% (3309/10,845) of which were caesareans. Among them, 1,953 were intrapartum primary caesarean deliveries. Eighty-four caesarean deliveries were performed in the second stage of labour. Of these, 13 cases were excluded from the study: 4 had high-risk pregnancies, 3 had a history of prior caesarean delivery, 3 had pregnancies in non-cephalic presentation, 2 had multiple pregnancies, and 1 had a pre-term pregnancy. There were no exclusions in the first-stage caesarean delivery group. The remaining 222 cases were analysed, consisting of 73 (32.8%) second-stage and 149 (67.2%) first-stage caesarean deliveries. There were neither participants who declined enrolment into the study nor participant dropouts. **Fig 1** illustrates the selection of cases.

**Table 1** displays maternal sociodemographic characteristics and pregnancy care. The age range of participants was 18–44 years, and there were no significant differences in maternal age or gestational age at delivery between the groups. Parity, occupation, and marital status were also comparable. Women who had a caesarean delivery in the second stage of labour had fewer antenatal care clinic attendances than those in the first-stage group. A significant difference in the level of education was observed between the groups. The majority of participants were married and had a post-secondary education.

**Table 1:** Maternal sociodemographic characteristics and pregnancy care in primary caesarean deliveries according to stage of labour.

Maternal characteristics	First-stage of labour (n = 149)				Second-stage of labour (n = 73)				P
	*M*	*SD*	*n*	*%*	*M*	*SD*	*n*	*%*	
**Maternal age(yrs)**	25.7	5.1			24.8	6			*0*.*234*
**Antenatal care clinic attendance**	5.3	1.4			4.7	1.3			***0*.*002***
**Gestational age**	39.6	1.3			40.2	1.4			*0*.*142*
**Birth weight (g)**	3335	444			3309	343			*0*.*643*
**Education**									
Primary			10	6.7			7	9.6	***0*.*019***
Secondary			48	32.2			36	49.3	
Tertiary			91	61.1			30	41.1	
**Parity**									
0			100	67.1			54	74	***0*.*168***
1–2			42	28.2			13	17.8	
>2			7	4.7			6	8.2	
**Occupation**									
Employed			18	12.1			7	9.6	*0*.*673*
Self-employed			29	19.5			11	15.1	
Student			32	21.5			20	27.4	
Unemployed			70	47			35	47.9	
**Marital status**									
Married			89	59.7			46	63	*0*.*638*
Single			60	40.3			27	37	
**History of pregnancy loss**									
No			139	93.3			71	97.3	*0*.*345*
Yes			10	6.7			2	2.7	

M, Mean; SD, Standard deviation.

Among women who underwent primary caesarean deliveries, the proportion of second-stage caesarean deliveries was 4.3% [95% CI: 2.9% - 4.7%] (84/1953).

Selected intrapartum characteristics are shown in **Table 2**.

**Table 2:** Intrapartum characteristics in primary caesarean deliveries according to stage of labour.

Intrapartum characteristics	First-stage of labour (n = 149)				Second-stage of labour (n = 73)				P
	*m*	*IQR*	*n*	*%*	*m*	*IQR*	*N*	*%*	
**Oxytocin augmentation**									
No			8	5.4			4	5.5	>0.99
Yes			141	94.6			69	94.5	
**Indication for caesarean delivery**									
Group 1: Non Progressive labour				89.9			65	89	*0*.*838*
Group 2: Fetal distress				10.1			8	11	
**Timing of operation**									
Day (08.00 to 17.59)			48	32.2			26	35.6	*0*.*613*
Night (18.00 to 07.59)			101	67.8			47	64.4	
**Skin incision to delivery time (min)**	4	*3*,*5*			4	3,6			*0*.*823*
**Total operation time(min)**	40	32,49			46	37,55			***0*.*002***
**Spinal anaesthesia**									
GA			4	2.7			0		*0*.*305*
Spinal			145	97.3			73	100	
**Delivery surgeon**									
Junior registrar (Y1/2)			80	53.7			36	49.3	*0*.*657*
Senior registrar (Y≥3)			68	45.6			36	49.3	
Consultant obstetrician			1	0.7			1	1.4	

GA, general anaesthesia.

### Adverse maternal outcomes

Second-stage caesarean delivery was associated with a greater risk of adverse maternal outcomes than first-stage labour (RR 3.272, 95% CI 2.45–4.50, P<0.001). Bladder injury occurred in 1.4% of second-stage caesarean deliveries and none in the first-stage group. Hysterectomy was rare, with 2.7% occurring in the second-stage group and none in the first-stage group. Primary PPH occurred in 9.6% of second-stage cases. Uterine extension occurred in eight cases, all in the second-stage group (11%). Second-stage caesarean delivery was associated with a higher risk of uterine atony (15.0% vs 3.4%, RR 2.13, 95% CI 1.99–3.98) and prolonged hospital stays (>3 days) (20.5% vs 0.1%, RR 5.65, 95% CI 1.29–7.77). Blood transfusion was also more likely with second-stage caesarean delivery (9.6% vs 0.1%, RR 2.44, 95% CI 1.86–4.44). These maternal complications are compared in **Table 3.**

**Table 3:** Adverse maternal outcomes in primary caesarean delivery.

Complications	Total no. of events	First-stage of labour n = 149		Second-stage of labour n = 73			
		n	(%)	n	(%)	RR	(95% CI)
**Atony**	16	5	3.4	11	15.0	2.13	1.99–3.98
**Adjacent tissue injury**	9	2	1.4	7	9.6	0.99	0.03–1.44
**Hysterectomy**	2	0	0	2	2.7	-	-
**Extension of uterine incision (T or J)**	8	0	0	8	11	-	-
**Bladder injury**	1	0	0	1	1.4	-	-
**Blood transfusion**	8	1	0.1	7	9.6	2.44	1.86–4.44
**Primary PPH**	7	0	0	7	9.6	-	-
**Length of postoperative stay > 3 days**	16	1	0.1	15	20.5	5.65	1.29–7.77
**Composite** *		7	15.4	26	35.6	3.272	2.28–4.71

*Atony, adjacent tissue injury, hysterectomy, bladder injury, uterine incision extension, primary post-partum haemorrhage, blood transfusion, length of postoperative hospital stay >3 days or in-hospital.

### Adverse perinatal outcomes

Women who had second-stage caesarean deliveries had higher rates of adverse perinatal outcome composites than those who had caesarean deliveries during the first-stage of labour (RR 2.748, 95% CI 1.97–3.84, P < 0.001), including neonatal trauma (1.4% vs 0%) and increased admission to the neonatal unit (RR 2.015, 95% CI 1.39–2.92). Second-stage caesarean delivery was also associated with lower 5-min APGAR scores (RR 2.64, 95% CI 1.87–3.72), but this may be due to prolonged labour and delivery duration rather than the procedure itself. Additionally, the risk of neonatal death was higher for women who underwent caesarean delivery in the second-stage of labour (RR 2.05, 95% CI 1.29–3.27) compared to those in the first-stage delivery group (**Table 4**).

**Table 4:** Comparison of adverse perinatal outcomes.

Complications	Total no. of events	First-stage of labour n = 149		Second-stage of labour n = 73			
		n	(%)	n	(%)	RR	(95% CI)
**5 min APGAR (≤3)**	14	3	2	11	15.1	2.636	1.87–3.72
**Neonatal trauma**	1	0	0	1	1.4	-	-
**Baby admitted into New Born Unit**	33	14	9.4	19	26	2.015	1.39–2.92
**Neonatal death**	15	6	4	9	12.3	2.054	1.29–3.27
**Composite** *		16	10.7	31	42.5	2.748	1.97–3.27

* Neonatal trauma, newborn unit admission, Apgar score ≤3 at 5min or, death within 24 hours of caesarean delivery.

## Discussion

The present study examined potential differences in adverse maternal and perinatal outcomes following caesarean delivery between the first and second stages of labour.

Women who had caesarean delivery during the second stage of labour had a 3.3-fold higher risk of maternal composite morbidity than those operated on during the first stage. The fetal head impaction into the maternal pelvis and an often oedematous lower uterine segment during prolonged labour may make caesarean delivery in the second stage challenging. In cephalic presentation, one of the most common manoeuvres for fetal head delivery involves slipping the surgeon’s hand into the uterine cavity and raising the head using fingers and palms through the uterine incision. This can cause tearing of the lower uterine segment and uterine vessel injury. The study demonstrated a lower maternal morbidity risk than a similar study in Turkey [10], possibly because of the inclusion of additional adverse events not investigated in the current study.

There was a higher occurrence of uterine incision extension during caesarean delivery in the second stage of labour (11% vs 0%). A US study by James M Alexander et al. reported significantly lower proportions of uterine incision extension during second-stage caesarean delivery, with only 0.4% of cases experiencing extension compared to 0.2% in the control group (P = 0.03) [11]. Almost all caesarean deliveries were performed by obstetrician gynaecologists in training, with junior registrars performing 49.3% of second-stage caesarean deliveries. While we did not examine the impact of obstetrics and gynaecology training on caesarean delivery outcomes, a survey of 150 obstetric trainees in the UK found that the majority agreed that training on second-stage caesarean deliveries would be beneficial. Specifically, 86% of registrars and 94% of senior house officers agreed, with two-thirds of registrars reporting that such training would increase confidence in managing a deeply impacted fetal head [12]. There is currently no institution or national protocol for second-stage caesarean delivery.

Compared to first-stage caesarean delivery, second-stage caesarean delivery was associated with a 2.13-fold higher risk of uterine atony following delivery (15% vs 3.4%, RR 2.13, 95% CI 1.99–3.98), possibly due to longer myometrium distension, oxytocin receptor desensitisation, and uterine muscle fatigue. This finding was consistent with a study by James Alexander et al. reporting atony rates of 9% vs 7% (P = 0.002) [11].

In second-stage caesarean deliveries, post-partum haemorrhage (PPH) i.e. blood loss ≥1000 mLs, occurred in 9.6% of cases, while none occurred in first-stage caesarean deliveries. All seven women with PPH required blood transfusion (9.6% vs 0.1%, RR 2.44, 95% CI 1.86–4.44). Uterine atony and injury to uterine blood vessels are the leading causes of PPH in caesarean delivery [13].

The occurrence of neonatal trauma after caesarean delivery during the second stage of labour compared to the first stage of labour was low (1.4% vs 0%). A study in Canada supported this finding [14]. However, a larger study by Asıcıoglu et al. demonstrated a significant increased risk of fetal injury following caesarean delivery in the second-stage of labour compared to the first-stage of labour, 6.7% vs 0.4%, RR 17.7, P < 0.01 [10].

Caesarean delivery in the second-stage of labour also increased the risk of NBU admission and neonatal death, likely due to increased fetal compromise with prolonged labour and delivery duration. Previous studies have shown mixed results regarding the risk of fetal asphyxia, with some reporting increased risk and others reporting no difference compared to first-stage caesarean delivery [10,14,15].

### Study strengths and limitations

Limitations of this study include that most outcomes were determined retrospectively, and data on the fetal delivery technique was missing in 90% of operation notes, making it impossible to evaluate outcomes based on delivery techniques. Additionally, assessments of abdominal palpation findings, asynclitism, caput, and moulding in the second stage of labour were not consistently documented, and reasons for prolonged second stage could not be inferred. However, the strengths of the study include a well-matched comparison group of first-stage caesarean deliveries and the inclusion of all second-stage primary caesarean deliveries within the study period, reducing sampling errors. This was also the first local study to compare morbidity and mortality between second-stage and first-stage caesarean deliveries.

## Conclusion

Compared to first-stage caesarean deliveries, second-stage deliveries were associated with a 3.3-fold higher risk of maternal morbidity, including intraoperative trauma, atony, blood transfusion, and a postoperative hospital stay of more than three days. Additionally, there was almost a threefold higher risk of adverse perinatal outcomes, including increased risk of a 5-min APGAR ≤3, admission to NBU, and neonatal death. Prevention and early identification of predictors for second-stage caesarean delivery can decrease these complications. Healthcare providers should be cognizant of these morbidities and have interventions prepared, particularly in resource-limited settings.

## Supporting information

S1 Checklist STROBE statement—checklist of items that should be included in reports of observational studies. (DOCX)

S1 Dataset (RAR)

## Abbreviations

ANC: Ante natal clinic
PPH: Postpartum haemorrhage
NBU: New Born Unit
MTRH: Moi Teaching and Referral hospital
RMBH: Riley Mother and Baby Hospital
