# Supplementary material for: Comparing adverse maternal and perinatal outcomes in primary caesarean delivery during first versus second-stage of labour in Kenya: An institution-based cohort study
Source: PLoS One. 2023 Nov 27;18(11):e0294266. doi: 10.1371/journal.pone.0294266 (PMC10681203; doi:10.1371/journal.pone.0294266)
Supplement: S1 Checklist — (DOCX) [file pone.0294266.s001.docx]

STROBE Statement—checklist of items that should be included in reports of observational studies

|  | Item No. | Recommendation | Page  No. | Relevant text from manuscript |
| --- | --- | --- | --- | --- |
| **Title and abstract** |  | (*a*) Indicate the study’s design with a commonly used term in the title or the abstract | 1 | Comparing adverse maternal and perinatal outcomes in primary caesarean delivery during first versus second-stage of labor: An institution-based cohort study. |
|  |  | (*b*) Provide in the abstract an informative and balanced summary of what was done and what was found | 1 |  |
| Introduction | | | |  |
| Background/rationale | 2 | Explain the scientific background and rationale for the investigation being reported | 2 | Second-stage caesareans may carry additional risk to both the mother and fetus due to fetal head impaction into the pelvis and the necessary manipulations required for delivery. However, data on outcomes and complications of this procedure from developing countries are limited. |
| Objectives | 3 | State specific objectives, including any prespecified hypotheses | 3 | To compare immediate adverse maternal and perinatal outcomes between second-stage and first-stage of labour |
| Methods | | | |  |
| Study design | 4 | Present key elements of study design early in the paper | 3 | hospital-based, cohort study |
| Setting | 5 | Describe the setting, locations, and relevant dates, including periods of recruitment, exposure, follow-up, and data collection | 3-4 | Setting: Riley Mother and Baby Hospital unit at the Moi Teaching and Referral Hospital/12 month study period/ second-stage caesarean deliveries (exposed of interest)/Follow up done till hospital discharge/ data abstraction form was used, data collected from hospital file records |
| Participants | 6 | (*a*) *Cohort study*—Give the eligibility criteria, and the sources and methods of selection of participants. Describe methods of follow-up  *Case-control study*—Give the eligibility criteria, and the sources and methods of case ascertainment and control selection. Give the rationale for the choice of cases and controls  *Cross-sectional study*—Give the eligibility criteria, and the sources and methods of selection of participants | 3-4 | Inclusion: singleton pregnancy, vertex presentation, ≥37+0 gestation, and with emergency primary caesarean delivery performed during active labor |
|  |  | (*b*) *Cohort study*—For matched studies, give matching criteria and number of exposed and unexposed  *Case-control study*—For matched studies, give matching criteria and the number of controls per case | 4-5 | Matching done by cesarean delivery indication category.  “Exposed” cases were compared with two ‘non-exposed’ participants per case considered representative of the cesarean delivery indication category. |
| Variables | 7 | Clearly define all outcomes, exposures, predictors, potential confounders, and effect modifiers. Give diagnostic criteria, if applicable | 3-6 | The composite adverse maternal outcome was defined as a woman experiencing any one or more of the following: intraoperative complications, primary postpartum haemorrhage, blood transfusion, Intensive care unit (ICU) admission or length of post-operative hospital stay >3 days. The composite adverse perinatal outcome was defined as a neonate experiencing any one or more of the following: neonatal trauma, New Born Unit (NBU) admission, Apgar score ≤7 at 5 min, or death within 24 hours of caesarean delivery.  The study's exclusion criteria were determined based on potential confounders |
| Data sources/ measurement | 8* | For each variable of interest, give sources of data and details of methods of assessment (measurement). Describe comparability of assessment methods if there is more than one group | 3 | A data abstraction form was used to collect the data. Participants’ hospital medical files were reviewed for intra-operative details, immediate post-operative neonatal and maternal outcomes. |
| Bias | 9 | Describe any efforts to address potential sources of bias | 3-6 | Matching done by cesarean delivery indication category.  “Exposed” cases were compared with 2 ‘non-exposed’  The study's exclusion criteria were determined based on potential confounders |
| Study size | 10 | Explain how the study size was arrived at | 3 | The study evaluated all eligible second-stage caesarean deliveries (exposed cases) carried out at the study site for 12 months’ |

Continued on next page

| Quantitative variables | 11 | Explain how quantitative variables were handled in the analyses. If applicable, describe which groupings were chosen and why | 5 |  |
| --- | --- | --- | --- | --- |
| Statistical methods | 12 | (*a*) Describe all statistical methods, including those used to control for confounding | 5 |  |
|  |  | (*b*) Describe any methods used to examine subgroups and interactions | 5 |  |
|  |  | (*c*) Explain how missing data were addressed | 5 |  |
|  |  | (*d*) *Cohort study*—If applicable, explain how loss to follow-up was addressed  *Case-control study*—If applicable, explain how matching of cases and controls was addressed  *Cross-sectional study*—If applicable, describe analytical methods taking account of sampling strategy | 5 | No loss to follow-up occurred in this study, likely due to the short follow-up period limited to only the hospital stay. |
|  |  | (*e*) Describe any sensitivity analyses |  |  |
| Results | | | | |
| Participants | 13* | (a) Report numbers of individuals at each stage of study—eg numbers potentially eligible, examined for eligibility, confirmed eligible, included in the study, completing follow-up, and analysed | 6 |  |
|  |  | (b) Give reasons for non-participation at each stage | 6 |  |
|  |  | (c) Consider use of a flow diagram | 6 |  |
| Descriptive data | 14* | (a) Give characteristics of study participants (eg demographic, clinical, social) and information on exposures and potential confounders | 6-7 |  |
|  |  | (b) Indicate number of participants with missing data for each variable of interest | N/A |  |
|  |  | (c) *Cohort study*—Summarise follow-up time (eg, average and total amount) | 6-7 |  |
| Outcome data | 15* | *Cohort study*—Report numbers of outcome events or summary measures over time | 5,9,10 | *Fig 1, Table 3, Table 4* |
|  |  | *Case-control study—*Report numbers in each exposure category, or summary measures of exposure |  |  |
|  |  | *Cross-sectional study—*Report numbers of outcome events or summary measures |  |  |
| Main results | 16 | (*a*) Give unadjusted estimates and, if applicable, confounder-adjusted estimates and their precision (eg, 95% confidence interval). Make clear which confounders were adjusted for and why they were included |  |  |
|  |  | (*b*) Report category boundaries when continuous variables were categorized |  |  |
|  |  | (*c*) If relevant, consider translating estimates of relative risk into absolute risk for a meaningful time period |  |  |

Continued on next page

| Other analyses | 17 | Report other analyses done—eg analyses of subgroups and interactions, and sensitivity analyses |  |  |
| --- | --- | --- | --- | --- |
| Discussion | | | | |
| Key results | 18 | Summarise key results with reference to study objectives | 12-15 |  |
| Limitations | 19 | Discuss limitations of the study, taking into account sources of potential bias or imprecision. Discuss both direction and magnitude of any potential bias | 15 |  |
| Interpretation | 20 | Give a cautious overall interpretation of results considering objectives, limitations, multiplicity of analyses, results from similar studies, and other relevant evidence | 15 |  |
| Generalisability | 21 | Discuss the generalisability (external validity) of the study results | 15 |  |
| Other information | |  | | |
| Funding | 22 | Give the source of funding and the role of the funders for the present study and, if applicable, for the original study on which the present article is based | 16 |  |
